# Temperature Variation Regulates the Trade-Off Between Pre- and Post-Hatching Investment in a Burying Beetle

**DOI:** 10.3390/insects16040378

**Published:** 2025-04-02

**Authors:** Donghui Ma, Long Ma, Jan Komdeur

**Affiliations:** 1 Groningen Institute for Evolutionary Life Sciences (GELIFES), University of Groningen, 9712 CP Groningen, The Netherlands; d.ma@rug.nl (D.M.); j.komdeur@rug.nl (J.K.); 2 GIGA-Neurosciences, Interdisciplinary Center for Biomedical Research (GIGA-Institute), University of Liège, 4000 Liège, Belgium

**Keywords:** ambient temperature, burying beetles, climate change, life history trade-offs, parental investment

## Abstract

Understanding how organisms respond to temperature variation is essential for assessing and predicting their resilience and vulnerability to environmental and climate changes. Here, using a biparental care burying beetle (*Nicrophorus vespilloides*), we tested whether and how parental investment in carcass preparation and ambient temperature interact to influence subsequent parental care behaviour and reproductive success. We employed a 3 × 2 factorial experiment, manipulating the levels of parental investment in carcass preparation (Reduced, Control, and Elevated) and ambient temperatures (benign: 20 °C and harsh: 23 °C) in breeding pairs. We found the following: (1) Irrespective of ambient temperature, males in the Reduced group decreased their pre-hatching care. (2) Across all investment groups, both sexes under higher temperature reduced post-hatching care. (3) Carcass-preparation investment and ambient temperature interactively influenced reproductive success. Overall, the harsh temperature decreased reproductive success. Furthermore, beetle pairs experiencing reduced carcass-preparation investment produced fewer eggs and lighter broods, while those experiencing elevated carcass-preparation investment produced smaller and lighter broods. Our findings provide new insights into how temperature variation affects parental investment strategies and enhance our understanding of the phenotypic plasticity in reproductive strategies that animals employ to cope with climate change.

## Simple Summary

Rising temperatures pose an unprecedented environmental challenge for organisms, with severe impacts on reproduction and survival. However, whether and how quickly adaptive behavioural adjustments in reproduction serve as an ecological response to thermal stress remain poorly understood. To address this gap, we used burying beetles (*Nicrophorus vespilloides*), which utilise carcasses as breeding resources and exhibit biparental care, to investigate how individual parental strategies change in response to varying levels of carcass-preparation investment (Reduced, Control, Elevated) under two ambient temperatures (benign: 20 °C vs. harsh: 23 °C). We found that reduced carcass-preparation investment led to decreased parental care before egg hatching in males but not in females, while this effect was independent of ambient temperature. Both males and females responded to the harsh temperature by decreasing their care during the larval stage, with negative effects of the harsh temperature on reproductive success. Interestingly, reduced or elevated investment in carcass preparation resulted in decreased reproductive success, when the ambient temperature was benign. These findings highlight temperature-dependent plasticity in parental investment across different reproductive stages, underscoring the role of parental care strategies in climate adaptation, particularly for species with complex parenting behaviours.

## 1. Introduction

Global warming, driven by climate change, is a major environmental and ecological challenge with profound impacts on biodiversity [1]. It severely affects individual survival and animal populations through both lethal and non-lethal mechanisms [2], such as temperature changes impairing animal reproduction [3,4,5,6]. Numerous studies across animal taxa have investigated the impacts of ambient temperature on life history traits, reproductive performance (e.g., sperm performance [7,8,9], fecundity [8,10], hatching success [11,12], offspring performance [13,14]), and behaviour (parental care [13,14,15]). These thermal effects may not only act directly on these traits but also indirectly by altering external factors that influence them. For example, rising temperatures and increased frequency of extreme thermal events can shift the phenology of plants and insects, which subsequently affects the timing of birds’ migration [16] and reproduction [17]. Alternatively, temperature changes can directly affect reproductive behaviours, such as the regulation of egg-laying time in great tits (*Parus major*) [18] and offspring fitness in tree swallows (*Tachycineta bicolor*) [19]. Importantly, the impacts of environmental temperature are often not limited to immediate effects but extend to long-term consequences as well [20]. For example, in the Chinese lacertid lizard (*Takydromus septentrionalis*), offspring of parents exposed to elevated ambient temperature exhibited higher survival rates under simulated future climate scenarios [21]. Another recent finding suggests that female burying beetles (*Nicrophorus vespilloides*) exposed to heatwaves during the pupal stage are more likely to provide enhanced direct care for their offspring [22]. Deciphering the thermal effects on individual behaviour and fitness could enhance our understanding of the evolutionary adaptation of animals to climate change.

Despite extensive research on the effects of thermal stress on reproductive success [23], the interactions between temperature and other ecological factors in shaping life history traits and behaviours remain less studied. Life history theory predicts trade-offs (i.e., negative relationship) between life history traits [24], such as offspring number versus offspring size [25] or current reproductive investment versus future survival and reproductive opportunities [26,27]. Parental investment involves the allocation of reproductive resources toward offspring at the current reproduction at the expense of future reproductive potential [28]. Such reproductive allocations are shaped by intrinsic factors (e.g., prior reproductive experience, energy reserves) and extrinsic conditions (e.g., resource availability, ambient temperature). Resource availability influences energy stores for parents, which in turn regulates trade-offs in parental investment between reproductive events [29,30]. Furthermore, ambient temperature modulates metabolic rates in both ectothermic and endothermic animals, with cascading effects on physiological processes and behaviours [31]. However, knowledge gaps remain regarding the impact of temperature variation on parental effort and its adjustment in specific periods during breeding. In this study, we investigate how parental investment (i.e., investment in processing resources) influences subsequent parental care behaviour and investment (both pre- and post-hatching) under different ambient temperatures in the burying beetles *N. vespilloides*.

Burying beetles (*Nicrophorus* spp.) are scavenger insects that exploit small vertebrate carcasses as both food and breeding resources. These beetles exhibit complex biparental care, with both males and females cooperatively engaging in carcass preparation and maintenance, brood defence, and food provisioning for offspring [32]. Parental care occurs in two phases: (1) pre-hatching, where beetles primarily process carcasses by removing any fur or feathers, rolling it into a ball, and smearing it with oral and anal secretions [33,34,35,36], and (2) post-hatching care, where they feed developing larvae with regurgitated and predigested food (i.e., direct care) and exhibit additional behaviours associated with carcass maintenance and defence (i.e., indirect care). Vertebrate carcasses are ephemeral and limited resources, making burying beetles opportunistic breeders that deploy flexible reproductive strategies in response to environmental changes [32]. Both male and female beetles differentially adjust their reproductive strategies based on various factors, such as age [37], inbreeding [38], brood size [39], nutritional status [40,41], carcass size [42], breeding patterns (i.e., cobreeding or communal breeding, pair breeding, and male and female uniparental breeding) [43,44,45,46,47,48], and temperature changes [6,13,15]. Some studies have demonstrated the impact of temperature variation on reproductive success and parental care behaviour in burying beetles [6,13,15,49]. For instance, in the burying beetle *N. orbicollis*, higher temperature significantly reduces egg number, fertilisation success, dispersed larval count, and mean larval mass, while parental care behaviours in both sexes remain unaffected [15]. However, whether and how temperature variation modulates parental investment and its trade-offs between the pre- and post-hatching period remain poorly understood.

To address this gap, we employed a 3 × 2 factorial experimental design to explore whether and how breeding pairs of beetles adjust their parental care behaviour and investment in response to variation in carcass-preparation effort within one breeding event, as well as how ambient temperature influences these parental adjustments. We manipulated three levels of parental investment in carcass preparation: reduced, control, and elevated treatments. In the reduced-investment group (Reduced group), beetle pairs were provided with prepared carcasses of mice, simulating low investment in carcass preparation for beetles. As the Control group, beetle pairs were provided newly thawed frozen carcasses of mice. To simulate a heightened-investment scenario, we also created the elevated-investment group (Elevated group), where beetle pairs have prepared carcasses before (i.e., with prior carcass preparation experience) and were subsequently given freshly thawed carcasses for breeding. Furthermore, beetles were exposed to two ambient temperatures (20 °C and 23 °C) throughout the whole period of breeding events. The 20 °C condition was considered benign, as the beetles used in this study were six-generation descendants reared under this temperature. In contrast, the 23 °C condition was deemed harsh, as it imposes reproductive costs on the beetles [6,15]. A pilot experiment demonstrated that 25 °C led to severe reproductive failure (0/120 broods), suggesting the 23 °C condition was stressful but not immediately detrimental to reproduction. To assess the effects of carcass-preparation investment and breeding temperature on individual care strategies and reproductive success, we measured pre- and post-hatching care behaviours, metrics of reproductive success (e.g., clutch size, brood size, brood mass, and offspring performance, i.e., mean larval mass). We also calculated the change in parental body mass to assess the parental performance after breeding.

Carcass preparation contains a series of energetically costly behaviours that may limit the parent’s ability to invest in their offspring [50,51], potentially reducing reproductive success. Additionally, the rate of carcass decomposition varies across ambient temperatures [52], which may lead to burying beetle parents adjusting their time and energy during carcass preparation, thereby affecting resource allocations for subsequent investments. For instance, under higher temperatures, parents may expend more effort on carcass preparation, limiting resources available for offspring care and reducing reproductive success. Thus, we predicted that beetles in the Reduced group would exhibit increased parental care and higher reproductive success, with this effect being more pronounced under benign temperatures. Conversely, we expected beetles in the Elevated group to show decreased parental care and lower reproductive success, whereas harsh temperatures may further exacerbate these negative effects on parental care and reproduction. Our study will illuminate how reproductive allocation within breeding events is influenced by temperature variations. By doing so, it will enhance our understanding of the phenotypic plasticity in parental investment strategies that animals, and insects in particular, potentially employ to adapt to the challenges posed by climate change.

## 2. Materials and Methods

### 2.1. Beetle Husbandry

All adult beetles in this study were sixth-generation descendants from an outbred laboratory population at the University of Groningen, originally sourced from a wild population in De Vosbergen, The Netherlands (53°08′ N, 06°35′ E), in the summer of 2022. Up to six same-sex adults from the same brood were housed together in clear rearing boxes (10 cm L × 6 cm W × 8 cm H) with moist peat and fed two beheaded mealworms (*Tenebrio molitor*) per beetle twice weekly. The husbandry room was set at 20 °C with a 16 h/8 h light cycle.

All fieldwork and laboratory procedures, including beetle collection and rearing, as well as the use of frozen mice, were designed and executed in accordance with the relevant institutional guidelines for the care and use of animals. Beetles were kept under optimal laboratory conditions, which ensured the high welfare of our beetle population. After completing all experiments, we maintained the beetles under the aforementioned conditions until their natural death.

### 2.2. Experimental Protocol

**Figure 1:**
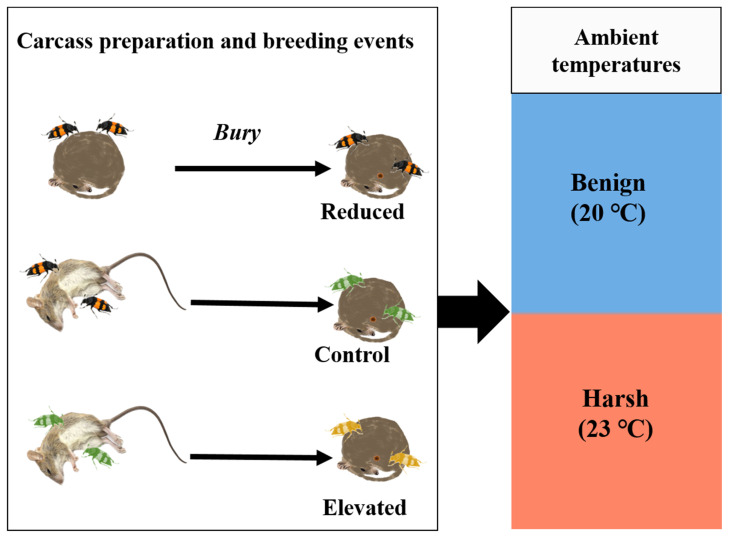
Schematic diagram of the experiment. On the left, three levels of investment in carcass preparation are shown: Reduced (virgin beetle pair bury one already prepared mouse carcass by another beetle pair and breed on it), Control (virgin beetle pair bury one newly thawed dead mouse carcass and breed on it), and Elevated (beetle pair that experienced carcass preparation once bury one newly thawed dead mouse carcass and breed on it). On the right, two ambient temperatures indicate the conditions for carcass preparation and breeding events. The beetle pairs without colouration (e.g., pairs in the Reduced group) do not engage in carcass preparation, while those with the green colouration prepare a carcass once, and those with the yellow colouration carry out the task twice.

We established three carcass-preparation investment groups (Reduced, Control, and Elevated) and conducted the experiment under two distinct ambient temperatures (20 °C and 23 °C) independently (Figure 1). In the Reduced group (20 °C: n = 30; 23 °C: n = 30), beetle pairs that did not undergo carcass preparation were provided with prepared carcasses of mice as breeding resources. In the Control group (20 °C: n = 63; 23 °C: n = 56), beetle pairs that did not undergo carcass preparation were provided freshly thawed dead mice as breeding resources. In the Elevated group (20 °C: n = 28; 23 °C: n = 25), beetle pairs that performed carcass preparation once were given freshly thawed dead mice as breeding resources.

Non-sibling, virgin, sexually mature (ca. 2 weeks post-eclosion) males (mean ± SE = 5.11 ± 0.02 mm) and size-matched females (mean ± SE = 5.05 ± 0.02 mm) were paired one day before initiating breeding. This pairing method minimised the potential effects of inbreeding [53], individual reproductive experiences [54], and parental body size [55] on reproductive success and ensured that females laid fertilised eggs. Prior to pairing, we measured each beetle’s body size (i.e., middle width of the pronotum) using an electric calliper (accuracy: 0.01 mm; Paget Trading Ltd., London, UK). Then, each pair of beetles was transferred into a breeding container (23 cm L × 19 cm W × 12.5 cm H) filled with 3–5 cm of moist peat and supplied a mouse carcass (prepared carcasses of mice for the Reduced group; thawed, unprepared carcasses for the Control and Elevated groups: mean ± SE = 23.71 ± 0.18 g) to initiate breeding. To obtain prepared carcasses for the Reduced groups, we conducted preliminary breeding trials before the main experiment. In these trials, beetle pairs were each given a carcass for preparation. After approximately 60 h, prepared carcasses (i.e., approximately 90% of fur was removed, and the body was shaped into a ball) were collected and stored at 4 °C for later use [54], whereas the beetle pairs that have prepared carcasses were transferred into new breeding containers for use in the Elevated groups. Specifically, during the carcass-preparation period (i.e., pre-hatching period, before egg hatching), beetle pairs in the Reduced group only need to drag the prepared carcass into the soil, bury it and add some oral and anal secretions to the carcass surface, but are released from hard work of removing the furs and rolling the carcass into a ball shape. Prior to initiating breeding events, we also weighed the body mass of beetles using an analytical balance (XS104, accuracy: 0.0001 g; Mettler-Toledo, LLC, Columbus, OH, USA). Additionally, all individuals were marked with small holes in the elytra (i.e., left one for males and right one for females) using a 00-insect pin to facilitate individual recognition during subsequent daily inspections.

After beetle pairs were given carcasses for breeding, we conducted visual inspections of the breeding containers three times daily (between 07:00 and 08:30 a.m., 1:30 and 3:00 pm, 8:00 and 10:30 p.m., at 5 h intervals) until breeding success, which is defined as at least one developing larva leaving the carcass (i.e., larval dispersal) [48]. At each inspection, we slightly removed the surface soil of the carcass to observe whether and which parent (s) was (were) present on or within the carcass. Parental presence was defined as parental care, no matter the actual behaviours of the parents (e.g., removing fur, rolling the carcass, feeding larvae, alert, cleaning the antennae and tarsus, and chewing carrion), while parental absence was defined as no parental care [45,48,54]. Before egg hatching, we also counted the number of visible eggs at the bottom of the breeding containers during daily inspections. The highest egg count observed during these checks was recorded as the clutch size [13,55,56]. At the larval dispersal stage, we counted all dispersed larvae to determine brood size and weighed them using an analytical balance (accuracy: 0.0001 g) to quantify brood mass, which provides estimates of reproductive success. Furthermore, we calculated mean larval mass, an estimate of offspring fitness, by dividing the brood mass by the brood size. Additionally, we measured the body mass of surviving parents at larval dispersal. The change in parental body mass during a breeding event was used as an indicator of residual reproductive value and calculated using the following formula: Body mass change = (Mass at larval dispersal − Mass before breeding)/Mass before breeding [45,48,54]. Breeding events without dispersed larvae or with dead parental individuals were excluded from our dataset.

Parental care across two reproductive stages—pre-hatching and post-hatching—was quantified separately using the following equations: (1) Pre-hatching care = (number of observations of the focal parent present on or within the carcass prior to the emergence of the first larva)/(total number of daily inspections conducted before larval emergence); (2) Post-hatching care = (number of observations of the focal parent present on or within the carcass after the emergence of the first larva)/(total number of daily inspections conducted after larval emergence). These calculations provide a comparative measure of parental investment during distinct developmental stages [45,48,54]. Meanwhile, parental care during the whole breeding phase was also calculated using the following equation: parental care = (number of observations of the focal parent present on or within the carcass)/(total number of daily inspections).

### 2.3. Statistical Analyses

All statistical analyses were performed in R version 4.3.1, with figures generated using the package ‘ggplot2’ (version 3.5.1) [57]. Generalized linear models (GLMs) were performed with the *glm* function in the ‘lme4’ package (version 1.1-34) [58] and *glm.nb* function in the ‘MASS’ package (version 7.3-60) [59]. Linear models (LMs) were performed with *lm* function. To assess the normality of model residuals, we used *simulateResiduals* function in the ‘DHARMa’ package (version 0.4.6) [60]. For all models, we reported likelihood ratios for the main effect and interaction between carcass-preparation investments and breeding temperatures using the *Anova* function in the ‘car’ package (version 3.1-2) [61]. When statistically significant effects were detected in the models (*p* < 0.05), we performed post hoc comparisons between the Control group and the other two treatment groups using the *emmeans* function with *trt.vs.ctrl* methods in the ‘emmeans’ package (version 1.7.1) [62].

Firstly, we employed binomial (logit link) GLMs to analyse male and female care separately for each stage, to investigate the effects of carcass-preparation investment and ambient temperature on parental care during pre-hatching and post-hatching periods. Secondly, to investigate the effect of parental care on reproductive success under our carcass-preparation investment treatments and temperatures, we used GLMs with either a Poisson or negative binomial distribution to analyse clutch size and brood size. After evaluating model fit using *stimulateResidules* function, we selected models with a negative binomial distribution as the best fit, and the statistical results for the terms in the final models were reported. Additionally, we used LMs to analyse brood mass. Thirdly, we used LMs to analyse mean larval mass to investigate the effect of parental investment on offspring performance. Finally, we used LMs to analyse parental body mass change to investigate the effect of parental investment on their residual reproductive value. All of the models included carcass-preparation investment, ambient temperature, their interaction, and carcass size as explanatory variables. Furthermore, parental body size was included as a covariate in the models for male and female care during the pre-hatching and post-hatching periods. Female body size and pre-hatching care were incorporated into the models for clutch size, while male and female parental care was included in the models for both brood size and brood mass. Additionally, parental body size and care were considered in the models analysing changes in parental body mass. We selected the best model for each response variable by discarding these explanatory variables that showed no significance, except for the carcass-preparation investment treatment and ambient temperature.

We conducted separate models for male and female pre- and post-hatching care, excluding their partner’s care as an explanatory variable. This approach was justified for three reasons. Firstly, our study did not aim to resolve the sexual conflict over parental care in relation to carcass-preparation investment and/or ambient temperature. Secondly, the sex-specific differences in care data distributions between pre- and post-hatching phases resulted in poor model convergence. Thirdly, incorporating higher-order interactions (e.g., carcass-preparation investment × ambient temperature × sex or carcass-preparation investment × partner’s care) introduced excessive complexity, obscuring biological interpretation.

## 3. Results

### 3.1. Effects of Carcass-Preparation Investment and Ambient Temperature on Parental Care

#### 3.1.1. Male Pre- and Post-Hatching Care

**Figure 2:**
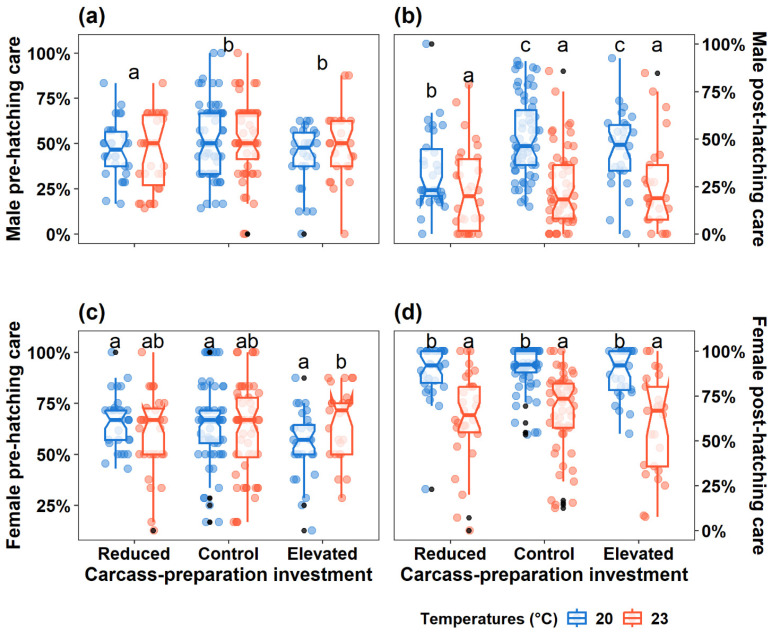
Effect of carcass-preparation investment and ambient temperature on male (**a**) pre-hatching and (**b**) post-hatching parental care, and female (**c**) pre-hatching and (**d**) post-hatching parental care. Boxplots show median, interquartile range, and minimum/maximum range. The coloured dots represent the real values of individual post-hatching care, while the black dots represent outliers. The letters in (**a**–**d**) indicate significant *trt.vs.ctrl* comparisons from the post hoc analysis, with which the same letter represents no difference. In (**a**), only the differences among these three treatments of carcass-preparation investment (i.e., Reduced vs. Control or Elevated vs. Control) were compared as the interaction of fixed factors did not show significance.

Carcass-preparation investment, but not ambient temperature, significantly influenced male pre-hatching care (Table 1). Compared to males in the Control group, those in the Reduced group exhibited significantly less pre-hatching care, while males in the Elevated group showed similar but slightly lower levels of pre-hatching care (Table 1, Figure 2a).

For post-hatching care, the interaction between carcass-preparation investment and ambient temperature was significant (Table 1). Specifically, (1) under the harsh temperature (at 23 °C), males exhibited reduced levels of post-hatching care across all carcass-preparation investment groups; (2) under the benign temperature (20 °C), males in the Reduced group exhibited less post-hatching care compared to those in the Control group; and (3) males in the Elevated and Control groups showed comparable levels of post-hatching care, regardless of temperature conditions (Table 1, Figure 2b).

#### 3.1.2. Female Pre- and Post-Hatching Care

The interaction between carcass-preparation investment and ambient temperature significantly affected female pre-hatching care (Table 1). Specifically, females in the Reduced and Control groups showed comparable levels of pre-hatching care under both temperatures, whereas in the Elevated group, females breeding under the harsh temperature showed more pre-hatching care than those breeding under the benign temperature (Table 1, Figure 2c). Across all groups, females exhibited comparable levels of pre-hatching care levels within the same temperature conditions (Table 1, Figure 2c).

For female post-hatching care, carcass-preparation investment had a marginal effect, while ambient temperature had a significant effect (Table 1). Regardless of carcass-preparation investment, females breeding under the harsh temperature exhibited reduced post-hatching care (Figure 2d).

### 3.2. Effects of Carcass-Preparation Investment and Ambient Temperature on Reproductive Success and Parental Body Mass Change

#### 3.2.1. Reproductive Success

**Figure 3:**
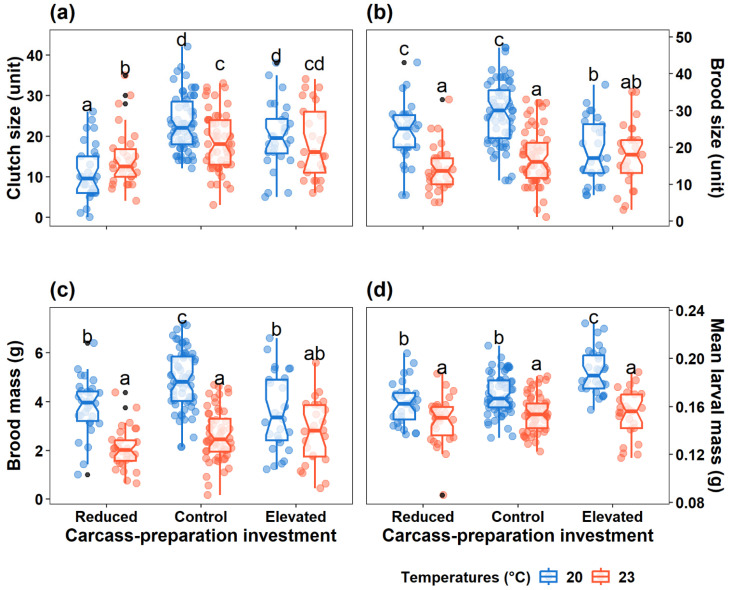
Effect of carcass-preparation investment and ambient temperature on (**a**) clutch size, (**b**) brood size, (**c**) brood mass, and (**d**) mean larval mass. Boxplots show median, interquartile range, and minimum/maximum range. The coloured dots represent the real values, while the black dots represent each treatment’s outliers. The letters indicate significant *trt.vs.ctrl* comparisons from the post hoc analysis, with which the same letter represents no difference.

The interaction between carcass-preparation investment and ambient temperature significantly affected clutch size (*LRχ*^2^ = 13.77, *p* = 0.001), brood size (*LRχ*^2^ = 10.94, *p* = 0.004), and brood mass (*F* = 5.74, *p* = 0.004). Specifically, under the harsh temperature, compared to the benign temperature, (1) the Reduced group produced more eggs per clutch but smaller and lighter broods, (2) the Control group produced fewer eggs, along with smaller and lighter broods, whereas (3) the Elevated group exhibited comparable clutch size, brood size, brood mass (Table 1 and Table 2; Figure 3a–c). Secondly, under the benign temperature, compared to the Control group, (1) the Reduced group produced fewer eggs, dispersed larvae of similar number and lighter broods; (2) the Elevated group produced comparable eggs, smaller and lighter broods (Table 2; Figure 3a–c). Thirdly, under the harsh temperature, (1) the Reduced groups produced fewer eggs than the Control group, while the Elevated group produced comparable eggs to the Control group; and (2) these groups produced broods of similar size and mass (Table 2; Figure 3a–c).

#### 3.2.2. Offspring Performance

The interaction between carcass-preparation investment and ambient temperature significantly affected mean larval mass (*F* = 6.58, *p* = 0.002). Specifically, (1) smaller dispersed larvae were produced under the harsh temperature (compared to the benign temperature) across all three groups; (2) under the benign temperature, larger dispersed larvae were produced in the Elevated group while dispersed larvae of similar mass were produced in the Reduced group, compared to the Control groups; and (3) dispersed larvae of similar mass were produced across the groups when beetle pairs bred under the harsh temperature (Table 2; Figure 3d).

#### 3.2.3. Parental Body Mass Change

**Figure 4:**
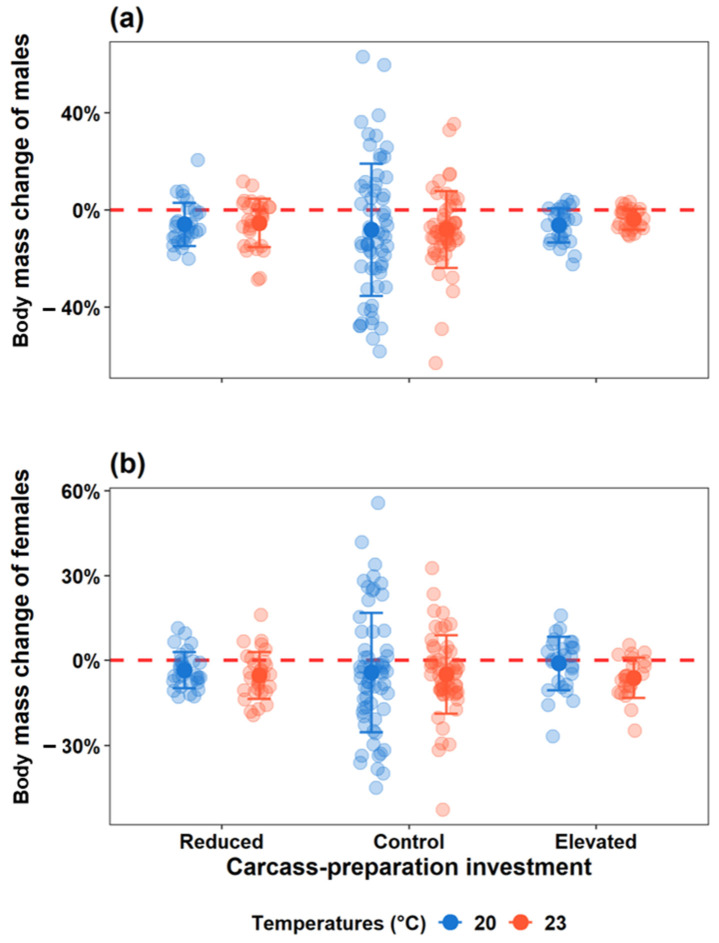
Effect of carcass-preparation investment and ambient temperature on (**a**) male and (**b**) female body mass change during breeding. The coloured dots represent the values of relative parental body mass change. Mean values are illustrated as dark-coloured dots with error bars. The red dashed line (y = 0) represents no body mass change during breeding.

Carcass-preparation investment, ambient temperature and their interaction had no significant effect on parental body mass change for either sex (*F* < 2.80, *p* > 0.1; Figure 4). However, smaller adults gained more or lost less body mass after breeding (male: *F* = 25.52, *p* < 0.001; female: *F* = 23.61, *p* < 0.001). Additionally, male but not female body mass change was positively correlated with parental care (*F* = 11.14, *p* < 0.001).

## 4. Discussion

Predicting how organisms behave in response to temperature variation driven by climate change is currently one of the hotspots in the field of ecology [63]. In this study, we tested the effects of ambient temperatures and “past parental investment” (i.e., parental investment in carcass preparation) on parental care strategies and reproductive success within breeding events in the burying beetles *N. vespilloides*. Our results revealed that males and females exhibit distinct parental investment across pre- and post-hatching periods depending on carcass-preparation investment and ambient temperature. Furthermore, the compounds of reproductive success were also significantly affected by the interaction of investment in carcass preparation and ambient temperature. Reduced investment in carcass preparation affected male parental care in both pre- and post-hatching periods but not female parental care. In contrast, elevated carcass-preparation investment had no significant effects on parental care for either sex. Additionally, harsh temperatures negatively impacted post-hatching care in both sexes but did not influence pre-hatching care, except for females in the Elevated group.

### 4.1. Reduced Carcass-Preparation Investment and Ambient Temperature Impact Parental Care and Reproductive Success

Contrary to our predictions, reduced carcass-preparation investment did not lead to increased parental care in either sex. Instead, males in the Reduced group exhibited lower levels of both the pre- and post-hatching care, whereas females maintained consistent levels of care. These findings partially align with a closely related study, where beetle individuals breeding on prepared carcasses provided less parental care for their broods and abandoned them earlier [54]. Previous research in *N. vespilloides*, along with others in the *Nicrophorus* genus, has demonstrated that males but not females show greater phenotypic plasticity in caregiving behaviour in response to shifts in external cues [15,47,48,56]. This sex-specific flexibility likely explains why only males adjusted their care in response to reduced investment in carcass preparation. In the Reduced group, breeding on prepared carcasses requires less time and energy in carcass preparation, bypassing the labour-intensive tasks of carcass preparation, such as fur removal, carcass shaping, and secretion for preservation. This was in line with what we found in the male pre-hatching care. Under such conditions, males could benefit from reducing their investment in carcass preparation, while females remain under selective pressure to maintain their caregiving levels to ensure reproductive success. This pattern aligns with the “Anisogamy” theory, which proposes that sexual asymmetry in reproductive investment arises from the energetic disparity in gamete production (i.e., egg production requiring more energy than sperm) [64]. This disparity might further extend to differences in parental investment during both pre- and post-natal stages of offspring development [28]. Previous research has suggested that female burying beetles provide care at their physical limitations [13,48,65], and our findings for female pre- and post-hatching care in the Reduced and Control groups further support the idea that females bear primary responsibility for offspring care throughout the breeding period (i.e., from preparing carcasses until larval dispersal).

When considering the effects of ambient temperature, our findings, partially aligning with the prediction, revealed that both males and females decreased their post-hatching care under harsh temperatures, regardless of the carcass-preparation investment being reduced or not. However, pre-hatching care remained unchanged across temperature conditions, suggesting that beetles may have reached a baseline investment threshold for carcass preparation to avoid reproductive failure. The observed reduction in post-hatching care under high temperatures is consistent with studies showing that increased metabolic costs under thermal stress reduce subsequent parental investment [6,13,15]. As ectothermic animals, the survival and reproductive activities of insects are profoundly influenced by ambient temperature [66], with which elevated temperature increases the metabolic costs of any physical activity or behaviour, including parental care (across pre- and post-hatching periods). In this study, the consistent individual pre-hatching care observed across the two temperature conditions (both in the Reduced and Control groups) may limit male and female energy allocation to post-hatching care. Furthermore, chronic exposure to high temperatures can cause negative effects on individual physiological performance [67,68]. Such impacts align with our findings, suggesting that thermal stress may play a critical role in shaping the observed reductions in individual post-hatching care.

The interactive effect of carcass-preparation investment and ambient temperature on reproductive success that we found aligns with our findings of the adjustment of parental care behaviour. In both the Reduced and Control groups, beetle pairs breeding under the harsh ambient temperature produced smaller and lighter broods with lower larval mass compared to those under benign temperature conditions, and this may be due to decreased investment in post-hatching care. This finding is consistent with previous studies on *Nicrophorus* species, which have documented adverse effects of elevated temperatures on reproductive outcomes [6,13,15,49,69,70]. One potential explanation is that thermal stress directly affects offspring development [67,68]. Additionally, higher temperatures may accelerate carcass decomposition, diminishing available resources for larvae [34]. This, in turn, explains why reproductive success did not differ between the Reduced and Control groups under harsh temperature conditions. This may reflect a baseline outcome driven by resource limitations affecting reproductive success.

Another interesting result is that females in the Reduced group laid fewer eggs than those in the Control group, whereas females in the Reduced group laid more eggs when they bred under the harsh temperature. In contrast, females in the Control group laid fewer eggs when they bred under the harsh temperature. These results are different from studies on *N. vespilloides*, which found no effect of heatwave on clutch size [6,13]. However, these findings partially align with studies on *N. nepalensis*, where higher ambient temperatures had a negative effect on clutch size [69]. The observed differences in clutch size may be explained by the influence of temperature and carcass-preparation conditions on ovarian development, as ovarian development is largely dependent on carcasses [30,71]. Prepared carcasses likely contain various pheromone profiles compared to fresh carcasses, potentially influencing ovarian maturation. Elevated temperatures may amplify these pheromonal effects, but further research is needed to confirm this hypothesis.

### 4.2. Elevated Carcass-Preparation Investment and Ambient Temperature Impact Parental Care and Reproductive Success

Contrary to our expectations, in both sexes of beetles, increased carcass-preparation investment did not affect parental care. Individuals in the Elevated group exhibited similar parental care levels compared to those in the Control group. One possible explanation is that the additional investment in carcass preparation may improve reproductive experience but may not impose significant energetic costs for individuals. On the one hand, beetle pairs in the Elevated group experienced ‘reproductive failure’, which may have influenced subsequent reproductive behaviour. On the other hand, these beetles may feed on the carcasses while burying them, thereby improving their body condition and offsetting their energy expenditures.

Aligning with our predictions, our results showed that, in response to high temperature, both males and females in the Elevated group decreased their post-hatching care. However, for pre-hatching care, such a response to ambient temperature is different between males and females. As with previous findings, both sexes decreased parental care levels under thermal stress [6,13]. This sex-specific shift in pre-hatching care suggests that females, but not males, adjust their investment based on prior reproductive experience, such as prior investment in carcass preparation. This contradicts some previous research that suggests males exhibit greater behavioural flexibility in reproduction and parental care [15,47,48,56]. Our results indicate that females, but not males, regulate their pre-hatching care in response to environmental challenges [65].

The interactive effects of carcass-preparation investment and ambient temperature on reproductive success are complex. Firstly, under the benign ambient temperature, beetles in the Elevated group produced comparable clutch sizes compared to those in the Control group, but their broods were smaller and lighter, and dispersed larvae were heavier. These results may be due to slightly reduced female pre-hatching care, leading to poorly prepared carcasses (20 °C: Control–Elevated, Est. ± SE = 0.36 ± 0.17, *z* = 2.17, *p* = 0.06), negatively impacting larval growth and survival. Additionally, prior ‘reproductive failure’ may have limited female energy reserves, reducing egg viability and larval survival. Under the harsh ambient temperature, there was no evidence that elevated carcass-preparation investment adversely affected reproductive success. This finding was consistent with observations of male and female pre- and post-hatching care under similar conditions, suggesting that higher ambient temperatures limited beetles’ flexibility in reproductive behaviours in response to variations in carcass-preparation investment. However, in the Elevated group, the reproductive success of beetles, excluding mean larval mass, was not adversely affected by the higher ambient temperatures. One possible explanation was that the increased female pre-hatching care (Elevated: 20–23 °C, Est. ± SE = −0.43 ± 0.20, *z* = −2.15, *p* = 0.03) resulted in well-prepared carcasses, mitigating the adverse effects of heat stress on reproductive success.

Finally, body mass changes in males and females were similar across all groups under both temperatures, indicating that neither carcass-preparation investment nor temperature significantly altered energy allocation during breeding. Body mass change reflects individual energy allocation during breeding, in which positive values indicate greater energy conservation to secure future reproductive opportunities, and negative values suggest higher investment in current broods [54]. Therefore, our findings indicated that, as a kind of opportunistic breeder, burying beetles consistently seize any available breeding opportunity, irrespective of external cues [32]. Furthermore, as shown in Figure 4, individuals in the Control group exhibited a greater difference in body mass change after breeding, particularly when bred under benign temperatures. This suggests that burying beetles are more inclined to allocate energy towards current reproduction rather than conserving energy for future reproductive opportunities when exposed to external stressors, such as increased ambient temperatures, pre-prepared carcasses, and prior reproductive failure, as shown in this study.

## 5. Conclusions

In the present study, we investigated whether and how ‘past investment’ (i.e., investment in carcass preparation) and ambient temperature influence parental care strategies in burying beetles across pre- and post-hatching periods. Our findings showed that males and females in breeding pairs adjusted their pre- and post-hatching care differently in response to our manipulations in carcass-preparation investments and temperatures, which may subsequently affect their reproductive success. Specifically, we found the following: (1) Irrespective of ambient temperatures, males in the Reduced group decreased their pre-hatching care. (2) Across all three carcass-preparation investment groups, both males and females breeding under the harsh temperature decreased their post-hatching care. (3) Overall, the harsh temperature decreased reproductive success, and beetle pairs in the Reduced group produced fewer eggs and lighter broods, while those in the Elevated group produced smaller and lighter broods. Our findings provide new insights into exploring how temperature variation affects organisms’ parental investment pattern and enhance our understanding of the phenotypic plasticity in reproductive strategies that animals employ to adapt to the challenges posed by climate change. However, due to the limitations of our experimental design and manipulations, we were unable to fully eliminate the influence of mates’ care when examining male and female care adjustments in response to carcass-preparation investment and temperature. For future research, we recommend more specific and accurate manipulations, such as establishing uni-female and uni-male parental care breeding events. Furthermore, temperature fluctuates over time in the real world; thus, it is valuable to conduct relevant projects in the future, under fluctuating rather than consistent ambient temperatures, to test how organisms behave.

## Figures and Tables

**Table 1:** The effects of carcass-preparation investment, ambient temperature, and their interaction on the pre- and post-hatching care of males and females, with the *trt.vs.ctrl* comparison results from the post hoc test for GLMs.

Variables	Male Pre-Hatching Care			Male Post-Hatching Care			Female Pre-Hatching Care			Female Post-Hatching Care	
	Est. ± SE	*LRχ²/z*	*p*	Est. ± SE	*LRχ²/z*	*p*	Est. ± SE	*LRχ²/z*	*p*	*LRχ²*	*p*
***1. Anova test for GLMs***											
Carcass-preparation investment		7.38	**0.02**		18.80	**<0.001**		0.78	0.68	5.65	0.06
Ambient temperature		0.19	0.67		131.39	**<0.001**		0.02	0.88	263.86	**<0.001**
Interaction					10.76	**0.005**		6.91	**0.03**		
***2. post-hoc test***											
Control-Reduced	0.28 ± 0.12	2.30	**0.04**								
Control-Elevated	0.26 ± 0.12	2.15	0.06								
Control: 20–23 °C				1.13 ± 0.11	9.84	**<0.001**	0.14 ± 0.15	0.97	0.33		
Reduced: 20–23 °C				0.48 ± 0.16	2.95	**0.003**	0.23 ± 0.20	1.15	0.25		
Elevated: 20–23 °C				0.91 ± 0.17	5.52	**<0.001**	−0.43 ± 0.20	−2.15	**0.03**		
20 °C: Control-Reduced				0.71 ± 0.13	5.33	**<0.001**	−0.05 ± 0.17	−0.31	0.94		
20 °C: Control-Elevated				0.21 ± 0.13	1.69	0.17	0.36 ± 0.17	2.17	0.06		
23 °C: Control-Reduced				0.06 ± 0.15	0.41	0.90	0.03 ± 0.18	0.19	0.98		
23 °C: Control-Elevated				−0.002 ± 0.16	−0.01	1.00	−0.21 ± 0.19	−1.15	0.44		

Note: Est. is the abbreviation of estimate. SE represents standard error.

**Table 2:** The *trt.vs.ctrl* comparison results from the post hoc test for GLMs on the components of reproductive success (clutch size, brood size, and brood mass) and offspring performance (mean larval mass).

Variables	Clutch Size			Brood Size			Brood Mass			Mean Larval Mass		
	Est. ± SE	*z*	*p*	Est. ± SE	*z*	*p*	Est. ± SE	*t*	*p*	Est. ± SE	*t*	*p*
Control: 20–23 °C	0.22 ± 0.07	2.91	**0.004**	0.23 ± 0.08	2.87	**0.004**	1.41 ± 0.24	5.89	**<0.001**	0.02 ± 0.003	4.50	**<0.001**
Reduced: 20–23 °C	−0.29 ± 0.11	−2.53	**0.01**	0.26 ± 0.10	2.52	**0.01**	1.00 ± 0.30	3.37	**0.001**	0.01 ± 0.005	3.15	**0.002**
Elevated: 20–23 °C	0.08 ± 0.11	0.71	0.48	−0.14 ± 0.11	−1.34	0.19	0.20 ± 0.31	0.63	0.53	0.04 ± 0.005	7.32	**<0.001**
20 °C: Control-Reduced	0.76 ± 0.10	7.80	**<0.001**	0.08 ± 0.08	0.93	0.58	0.61 ± 0.25	2.41	**0.03**	0.008 ± 0.004	1.94	0.11
20 °C: Control-Elevated	0.15 ± 0.09	1.59	0.21	0.32 ± 0.08	3.90	**<0.001**	1.03 ± 0.25	4.12	**<0.001**	−0.019 ± 0.004	−4.71	**<0.001**
23 °C: Control-Reduced	0.25 ± 0.10	2.63	**0.02**	0.11± 0.09	1.16	0.43	0.20 ± 0.26	0.79	0.68	0.006 ± 0.004	1.43	0.28
23 °C: Control-Elevated	0.009 ± 0.10	0.09	0.99	−0.05 ± 0.09	−0.54	0.83	−0.19 ± 0.26	−0.72	0.72	0.0005 ± 0.004	0.11	0.99

Note: Est. is the abbreviation of estimate. SE represents standard error.

## Data Availability

The original data presented in the study are openly available in Zenodo at [DOI: 10.5281/zenodo.14943887].
